# Advertisements

**Published:** 1858-09

**Authors:** 


### ADVERTISEMENTS.

We desire to call attention to the advertisements of the "New Orleans School of Medicine" and the "University of Louisville," contained in our last number, and to that of the "Virginia Medical College" at Richmond, Va., contained in the present.

These are all schools of decided merit, and in either of them will be found ample facilities for obtaining a complete medical education.

